# Spontaneous Retroperitoneal Bleeding as a Complication of Unusual Renal Paraganglioma

**DOI:** 10.1155/2022/6882451

**Published:** 2022-08-30

**Authors:** Tawfeeq I. Sangey, Hamim Abdul Rusheke, Ansaar I. Sangey, Nimishkumar Chaya, Advera Ngaiza

**Affiliations:** ^1^TMJ Super Specialized Polyclinic, Dar Es Salaam, Tanzania; ^2^School of Medicine and Dentistry, University of Dodoma, Dodoma, Tanzania; ^3^Muhimbili National Hospital, Dar Es Salaam, Tanzania; ^4^TMJ Hospital, Dar Es Salaam, Tanzania

## Abstract

Spontaneous retroperitoneal bleeding is a rare occurrence that might have catastrophic implications. We present a 58-year-old male with a 4-day history of progressively worsening left-sided flank pain due to retroperitoneal hemorrhage from a nonfunctional renal paraganglioma. Abdominal contrast CT scan was helpful in locating the tumor, estimating tumor size and extent of bleeding, visualizing the interaction between the tumor and the surroundings, and ruling out any potential metastasis; however, it lacked specificity in identifying the origin of the mass, needing histologic investigation for a conclusive diagnosis. MRI was not available at our center. We report a rare case of spontaneous retroperitoneal bleeding as a complication of an unusual nonfunctional renal paraganglioma, which was initially misdiagnosed as renal cell carcinoma but later confirmed by postoperative histopathology.

## 1. Introduction

Extra-adrenal autonomic paragangliomas are rare neuroendocrine tumors that develop from the paraganglia. These are tiny organs made up primarily of neuroendocrine cells generated from the embryonic neural crest that can release catecholamines [1]. Paragangliomas are closely related to pheochromocytomas, and their incident rate is about 0.6 cases per 100,000 person-years [2, 3]. In 2017, the World Health Organization described the term “paraganglioma” for extra-adrenal tumors, while the term “pheochromocytoma” was restricted to adrenal origin [4]. Histologic findings are insufficient to distinguish these two tumor types; anatomical location is employed to do so [5]. The majority of paragangliomas are nonhypersecretory and are found in the head and neck as compared to the abdominal ones [6]. The kidney, bladder, and mediastinum are among the common intra-abdominal sites [7–9]. Based on the ability of catecholamine secretion, paragangliomas can be classified as functional or nonfunctional [6, 10]. Functioning tumors present with myriad symptoms such as hypertension, headaches, diaphoresis, palpitations, and tremor [6]. Intra-abdominal paragangliomas have been linked to catecholamine hypersecretion and have been found to have the classical triad of symptoms and hypertension, as well as being benign tumors [6, 11]. However, with the increasing use of high-resolution imaging techniques in the past decades more incidentalomas have been detected, but they lack specificity due to their variable imaging appearance; surgical resection may be the only way to reach a diagnosis. Histopathology and immunohistochemistry of the excised tumor are used to make the final diagnosis [12, 13].

Spontaneous retroperitoneal hemorrhage is a well-known but uncommon condition characterized by retroperitoneal bleeding without a prior history of trauma. It may strike fast and quietly, with catastrophic consequences if not diagnosed and treated on time. Clinically, it presents with acute abdominal pain, mainly in the affected flank, palpable mass, and hypovolemic shock in severe blood loss [14]. Spontaneous retroperitoneal hemorrhage from the kidney is a rare entity. The most common renal cause is angiomyolipoma and renal cell carcinoma [15, 16]. Hemorrhagic renal paraganglioma is an extremely rare entity, and to the best of our knowledge, it has not been previously reported.

## 2. Case Report

A 58-year-old man presented to the emergency department complaining of left-side abdominal pain for four days. The pain began suddenly and moved to the left groin and testes. However, it was only associated with constipation through passing flatus. There was no history of vomiting, sweating, palpitation, or hematuria. There was no past medical history of hypertension or diabetes.

On examination, the patient was pale (anemic), had no pedal oedema, and had normal blood pressure of 120/70 mm/Hg. There were distended abdomen and tenderness at the left hypochondrium, left lumbar fossa, and suprapubic region.

Hematology parameters were as follows: HB, 6.0 g/dl (13.0–18.0); leukocytes, 16.47 (4.0–12.0); platelets, 170 × 10(9) (150–450); ESR, 38 mm/hr [1–45]; creatinine, 143 umol/l (62–106); eGFR, 46 ml/min; sodium, 132 mmol/l (136–145); potassium, 4.3 mmol/l (3.5–5.1); and stool for *H. pylori*, negative; stool occult blood test, negative; and urine analysis, no detection of erythrocytes.

On radiological examination, ultrasound showed a left renal complex lesion that is predominantly solid with some cystic components seen at the left upper renal pole. It demonstrated mild internal vascularity on the Doppler study.

**Figure 1:**
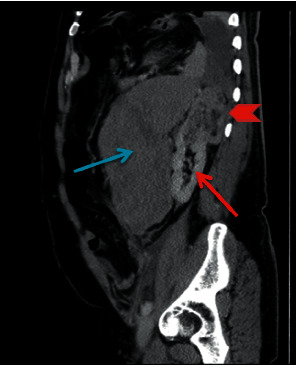
Contrast sagittal section shows a large hematoma at the left retroperitoneal region (blue arrow), the normal-appearing left kidney (thin red arrow), and an exophytic left upper pole renal mass (red arrowhead).

**Figure 2:**
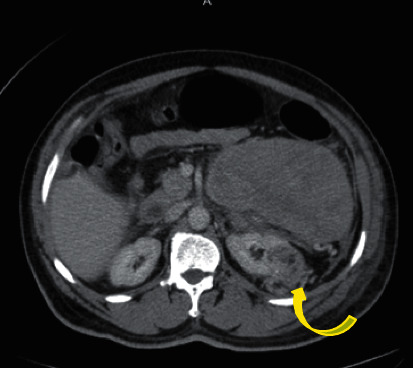
Contrast axial view section shows an ill-defined margin between the exophytic mass and the kidney (curved yellow arrow).

**Figure 3:**
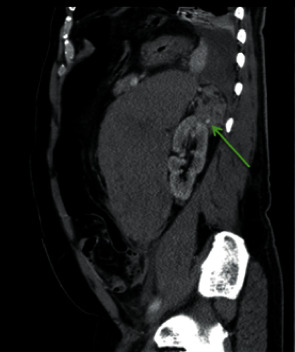
Green arrow shows a contrast extravasation consistent with active bleeding.

Subsequent computed tomography (CT) of abdomen and pelvis showed an exophytic upper pole cortical mass (red arrowhead) in the left kidney (red arrow) measuring 7 × 4.5 × 7.3 cm in transverse, anterior-posterior, and craniocaudal diameter, respectively (Figure 1). On contrast study, the mass was characterized by heterogeneous enhancement with solid and cystic components. An axial image showed an ill-defined margin between the mass and the kidney denoted by a curved yellow arrow (Figure 2). A focal rounded area of intense enhancement (green arrow) was seen on arterial phase images consistent with active low-intensity bleeding (Figure 3). On noncontrast study, no calcification was seen. There was a large retroperitoneal hematoma, which was isodense to the muscle located anterior to the left kidney (blue arrow). There was no sign of local invasion into the adjacent structure. No evidence of perirenal retroperitoneal lymph nodes or bony lesions was seen. Bilateral adrenal glands were reported as normal. A provisional diagnosis of spontaneous hematoma due to bleeding renal cell carcinoma was made.

**Figure 4:**
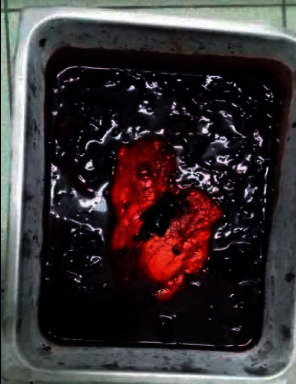
Postoperative left nephrectomy specimen with evacuated 3.5 L blood and clots.

**Figure 5:**
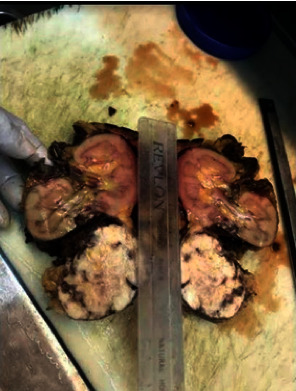
Gross anatomy specimen showing the exophytic renal cortical tumor incontinuity with the renal cortex (black arrow).

The patient was then scheduled for surgery, which included a left nephrectomy with complete mass excision, and a total of 3.5 liters of blood and clots were removed from the peritoneum (Figure 4). The hilum was sealed, and hemostasis was achieved. No intraoperative complication occurred. The excised mass was then sent for histopathologic evaluation. The patient was discharged after 3 days of stay in hospital without any postoperative complications. Gross anatomy specimen is kidney with attached tumor size of 7 cm (Figure 5). Tumor had a bright white tissue (about half) and a dirty pinkish white tissue (other half).

**Figure 6:**
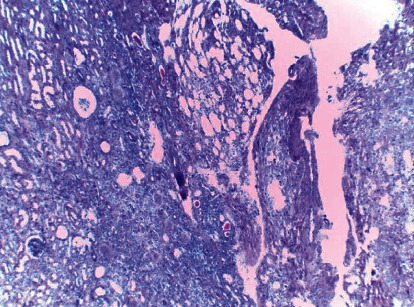
On low magnification, the left side shows normal-looking renal tubules and the right side shows the fatty tissue with round blue cells consistent with tumor cells.

**Figure 7:**
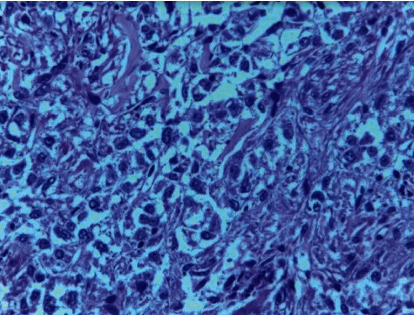
On high magnification, remarkable cellularity, granular cytoplasm, and central or slightly eccentric nuclei are observed. There are no abnormal mitosis and mild nuclear pleomorphism.

Histology: on low magnification, the left side shows normal-looking renal tubules and the right side shows the fatty tissue with round blue cells consistent with tumor cells (Figure 6). On high magnification, proliferating round cells with central nuclei are found. Septa separate them into vague groups and vague gland-like formations (nesting and trabecular pattern). Tumor cells had round-to-oval nuclei with abundant granular cytoplasm. Mitotic figures were not seen. Pericapsular fat shows round blue tumor cells. Cellularity is moderate. No cellular spindling or necrosis was seen (Figure 7). Ki67 activity was less than 1%. A diagnosis of pheochromocytoma was found on a conventional HE stain. PASS score was 6/21 (benign or low-grade malignant behaviour possible). A stronger scoring system (GAPP) is required to determine malignant potential, which mostly requires a combination of histologic, somatic molecular, and clinical data (urinary metanephrine/nonmetanephrine levels); the later was unfortunately not performed in our case.

## 3. Discussion

Paragangliomas/pheochromocytoma are rare neuroendocrine tumors arising from chromaffin cell tumors located at any extra-adrenal site along the sympathetic or parasympathetic nervous system and account for 15 to 20% of chromaffin cell tumors [1, 8]. The incidence of extra-adrenal paragangliomas is estimated to be 2–8 per million individuals and has been significantly increasing over the past two decades [17]. They can occur at any age ranging from 16 to 84 years at the time of diagnosis though they are commonly found in young adults with a median age of 30–43 years and frequently in female patients [18, 19]. At least 30% of the patients have been shown to have certain hereditary cancer-predisposing disorders, such as multiple endocrine neoplasia, neurofibromatosis type 1, or von Hippel–Lindau syndrome [20].

Retroperitoneal extra-adrenal paragangliomas make up the majority of extra-adrenal paragangliomas, accounting for 85% of all extra-adrenal paragangliomas [21]. The renal pelvis (4.9%) is the third most common site of paragangliomas in the genitourinary tract, after the bladder (79.2%) and urethra (12.7%) [22]. Kidney paragangliomas are exceedingly rare and may develop in the hilum (32%), upper pole (26%), or lower pole (42%) within the kidney [23].

Diagnosis of paraganglioma necessitates the combination of clinical symptoms, biochemical evaluation of catecholamine production by the tumor, and tumor location [6, 24]. The patients' clinical picture can be variable from asymptomatic to a dramatic life-threatening event. The classical triad consists of headache, palpitation, and diaphoresis together with hypertension, where a diagnosis of pheochromocytoma/paraganglioma should be suspected [6] The classical triad is in fact nowadays not that common; according to a study conducted by Kopetschke, the typical triad of symptoms was found in only 10% of cases. 6.1% were normotensive, while 10% were asymptomatic [25]. Hypertension has also been linked to tumors located in the renal pelvis and hilum due to renal artery stenosis caused by the local mass effect [26]. Few studies reported paraganglioma crisis with severe life-threatening complications including cardiovascular emergencies and hypovolemic shock due to severe tumor bleeding [27–29].

**Figure 8:**
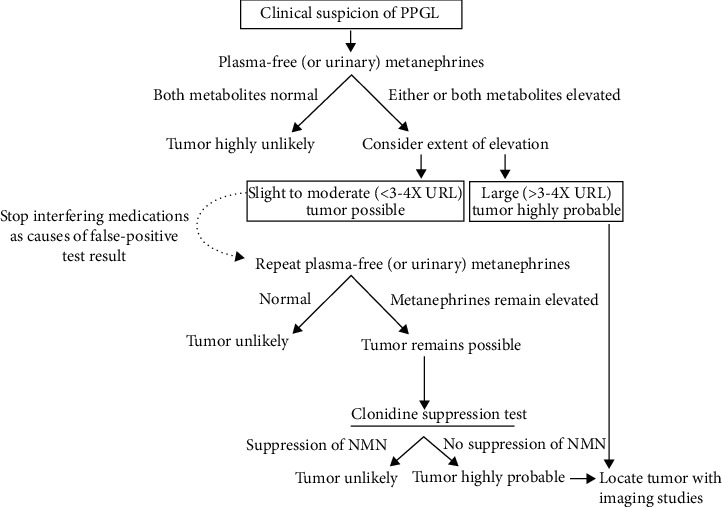
Flow chart illustrating the indications for the biochemical screening algorithm in a clinically suspected paraganglioma. NMN: normetanephrine. Citation: European Journal of Endocrinology 170, 3; 10.1530/EJE-13-0882.

The biochemical diagnosis of hyperfunctional paraganglioma or adrenal pheochromocytoma is based on excessive excretion of catecholamines and their metabolites. The 24 h urinary excretion of total metanephrine and catecholamine metabolites was determined to have a sensitivity of 74% and 89.9%, respectively [6]. The Endocrine Society Clinical Guidelines Subcommittee has also established biochemical testing recommendations for the diagnosis of paraganglioma; measurements of plasma-free or urine-fractionated metanephrines should be included in the first biochemical testing for paragangliomas. Preanalytical variables that contribute to false-positive or false-negative findings should be considered [30]. A simplified flow chart explaining the grounds for a biochemical screening procedure in a clinically suspected paraganglioma is shown in Figure 8. A threefold to fourfold increase in plasma-free normetanephrine or plasma-free metanephrine over the upper limits of the adult reference intervals indicates the likely existence of paraganglioma.

As per our case, preoperative screening for catecholamines was regrettably not performed because the patient initially presented with acute abdomen and severe anemia instead of catecholamine-related symptoms and was also normotensive. In biochemically silent abdominal paragangliomas (nonfunctional), the lack of catecholamine secretion is not related to catecholamine storage or secretion mechanisms, but rather to a deficiency in catecholamine synthesis that results in a near-complete lack of releasable reserves. The problem is caused by a lack of tyrosine hydroxylase [12, 31]. And because of the absence of clinical symptoms, nonfunctional paragangliomas are frequently misdiagnosed, posing a significant diagnostic challenge [32].

Incidental discovery of nonfunctional paragangliomas has increased over the last two decades using high-resolution abdominal imaging techniques prior to the onset of symptoms. 29.4% of the 201 total study population was diagnosed incidentally to have pheochromocytoma/paraganglioma [25]. Imaging is widely used to indicate tumor location, assess regional spread or multifocality, and exclude metastasis [33]. Paragangliomas have no pathognomonic imaging characteristics and demonstrate imaging features similar to pheochromocytomas. Enhancement characteristics on MRI are similar to CT, as Paragangliomas demonstrate rapid, avid enhancement and delayed washout, but up to one-third of pheochromocytomas have overlapping washout characteristics with adrenal adenomas [34]. A cutoff CT density of less than 10 Hounsfield units (HU) provides 100% sensitivity for diagnosing an adrenal adenoma, with a relatively low specificity of 40.5% [35]. Cystic changes, necrosis, and internal calcifications are commonly described in pheochromocytoma/paragangliomas. Internal hemorrhage can sometimes be seen within the tumor. Calcifications have been described in 10–12% of cases of pheochromocytomas and are better appreciated on noncontrast CT [36]. Pheochromocytomas/paragangliomas have a significantly more spherical shape, a sharp central necrosis, and a ring sign having a high sensitivity and specificity of 80% and 95%, respectively. An unsharp necrosis was presented as poorly defined and with a blurry margin [37]. On CT, a small size paraganglioma/pheochromocytoma is often misdiagnosed as renal cell carcinoma [23, 36] as was misdiagnosed in our case.

The classical “salt-and-pepper” appearance of paragangliomas on T1- and T2-weighted images is due to the presence of punctate areas of low signal intensity corresponding to flow voids from tumor vascularity, and hyperintense signal corresponding to areas of hemorrhage in the tumor. This sign has been described more in head and neck paragangliomas than in trunk paragangliomas, and it is also neither sensitive nor specific, as it can be seen in any hypervascularized tumor, specifically angiofibromas [38].

Spontaneous rupture of nonfunctioning retroperitoneal paraganglioma is rare and quite difficult to be diagnosed initially as it presents with severe abdominal pain that mimics other acute surgical conditions [39, 40]. Our patient was also asymptomatic until the acute abdomen discomfort caused by hemorrhagic retroperitoneal paraganglioma.

Spontaneous hemorrhage induced by paraganglioma can present as an abdominal catastrophe, which tends to be lethal. In a review of literature, so far 50–53 cases have been identified with ruptured pheochromocytoma worldwide and mortality rate of 32–34% globally [41, 42]. Although the pathophysiology of spontaneous rupture of a nonfunctioning paraganglioma is unknown, it may be related to a number of factors, such as the tumor's highly vascular nature and its potential for precarious microcirculation due to high levels of tissue vasoconstrictor substances, which could lead to spontaneously large intratumoral bleeding [43].

Histologically, paraganglioma is usually composed of solid nests known as “zellballen” of round-to-oval or elongated cells with abundant granular amphophilic or basophilic cytoplasm, within a vascular stroma, but they may also have acidophilic cytoplasm [24]. Malignant potential depends on the tumor size, level of excess catecholamine secretion, distance metastasize at time of primary tumor detection, or susceptibility to gene mutation. No cellular characteristic changes are noted in patients with a benign compared to those with a malignant tumor [42].

A concept of risk stratification is used to describe the metastatic potential of pheochromocytoma and paraganglioma [20]. Malignant pheochromocytoma has a more aggressive course than malignant paraganglioma in terms of long-term survival outcome [44] kim et al. reported 9.0% of patients with pheochromocytoma and 20.5% of those with paraganglioma developed metastatic lesions during the median 38.0 months of follow-up after primary surgical resection [45]. In 2002, Thompson designed the Pheochromocytoma of the Adrenal Gland Scaled Score (PASS) chart incorporating histologic features such as capsular or vascular invasion, mitotic rate, and necrosis [46]. Over significant possible interobserver variability, Kimura et al. introduced an alternative score chart, the Grading system for Adrenal Pheochromocytoma and Paraganglioma (GAPP), which integrates histopathologic, biochemical, and clinical data. Its scoring system is composed of six parameters that have been considered prognostic factors: histologic pattern, cellularity, comedo necrosis, capsular/vascular invasion, Ki67 labeling index, and catecholamine phenotype, with a total of 10 possible points (Table 1). A score of 0–2 is well, 3–6 is moderately, and 7–10 is poorly differentiated types (Table 2). It also has an advantage of predicting metastasize and clinical prognostication [20].

Complete surgical excision is the treatment of choice for extra-adrenal retroperitoneal paragangliomas, and recurrent or metastatic disease but should be carried out on the basis of correct drug preparation of *α*-receptor blocker prior to surgery [47]. In the literature, hypertension was the most common encountered as a perianesthetic risk in 31% of the 143 patients [48]; this can pose a high risk of developing intraoperative cardiac emergencies. There is also an increased risk of intraoperative massive blood loss in subjects with tumors proximal to vessels or other organs, with tumors ≥5 cm in size, or with preoperative preparation time of less than 14 days [49]. There was no intraoperative complication seen in our case study.

Furthermore, risk stratification is required to tailor the follow-up protocol after complete resection of paraganglioma. The overall rate of recurrent disease was found to be 0.98 events/100 person-years, and primary tumors larger than 5 cm have a higher risk of recurrence [50].

## 4. Conclusion

Retroperitoneal pheochromocytoma/paragangliomas are rare tumors that are usually benign with good prognosis. The clinical picture of the patient might range from asymptomatic to a dramatic life-threatening occurrence such as sudden acute bleeding from the tumor. Incidental discovery of the pheochromocytoma and paragangliomas on imaging is becoming an increasingly essential mode of their diagnosis. Radiological differential diagnoses are often inadequate or challenging in such cases, especially for tumors originating from kidneys. Therefore, it is important for clinicians and radiologists to be more aware of pheochromocytoma/paragangliomas and their various appearances in order to avoid potential surgical complications without further evaluation prior.

## Figures and Tables

**Table 1:** Parameters and score in the Grading system for Adrenal Pheochromocytoma and Paraganglioma (GAPP) by Noriko Kimura et al. (https://doi.org/10.3390/jcm7090242).

Parameters	Score
*Histologic pattern*	
Zellballen	0
Large and irregular cell nest	1
Pseudorosette (even focal)	1

*Cellularity*	
Low (less than 150 cells/U^*∗*^)	0
Moderate (150–250 cells/U^*∗*^)	1
High (more than 250 cells/U^*∗*^)	2

*Comedo necrosis*	
Absence	0
Presence	2

*Vascular or capsular invasion*	
Absence	0
Presence	1

*Ki67 labeling index*	
Less than 1%	0
1–3%	1
More than 3%	2

*Catecholamine type*	
Adrenaline type (A^*∗∗*^, or a + NA^*∗∗∗*^)	0
Noradrenaline type (NA, or NA + DA^*∗∗∗∗*^)	1
Nonfunctioning type	0

**Total maximum score**	10

U^*∗*^: cells in the unit of 10 mm under high--power field (400); A^*∗∗*^: adrenaline; NE^*∗∗∗*^: noradrenaline; DA^*∗∗∗∗*^: dopamine.

**Table 2:** GAPP score and risk stratification by Noriko Kimura et al. (https://doi.org/10.3390/jcm7090242).

Total score (points)	Histologic grade (frequency)	Metastatic rate	5-year survival (%)	Risk stratification
0–2	Well differentiated (68%)	3.6%	100	Low
3–6	Moderately differentiated (22%)	60.0%	66.8	Intermediate
7–10	Poorly differentiated (10%)	88.2%	22.4	High
